# A New Test for Achilles Tendinopathy Based on Kager’s Fat Pad Clinical Assessment Predictive Values

**DOI:** 10.3390/jcm12165183

**Published:** 2023-08-09

**Authors:** David Rodríguez-Sanz, Marta Elena Losa-Iglesias, Ricardo Becerro de Bengoa-Vallejo, Zacarías Sánchez-Milá, Hend Adel Abdelhalim Dorgham, Ahmed Ebrahim Elerian, Tian Yu, César Calvo-Lobo, Jorge Velázquez-Saornil, Eva María Martínez Jimene

**Affiliations:** 1 Faculty of Nursing, Physiotherapy and Podiatry, Universidad Complutense de Madrid, 28040 Madrid, Spain; ribebeva@ucm.es (R.B.d.B.-V.); tina.yu1234@icloud.com (T.Y.); cescalvo@ucm.es (C.C.-L.); evamam03@ucm.es (E.M.M.J.); 2 Faculty of Health Sciences, Universidad Rey Juan Carlos, 28933 Móstoles, Spain; marta.losa@urjc.es (M.E.L.-I.); jorge.velazquezsaornil@ucavila.es (J.V.-S.); 3 Department of Physiotherapy, Faculty of Health Sciences, Universidad Católica de Ávila, 05005 Ávila, Spain; zacarias.sanchez@ucavila.es; 4 Department of Physical Therapy, Ras Eltein General Hospital, Alexandria 21513, Egypt; drhenddorgham@rocketmail.com; 5 Department of Basic Science for Physical Therapy, Faculty of Physical Therapy, Al Salam University, Tanta 31511, Egypt; dr_ahmed_elerian77@yahoo.com

**Keywords:** Achilles tendon, ultrasound, test

## Abstract

Background This study aimed to check the diagnostic accuracy of a new test to identify Achilles tendinopathy. Study Design: Observational study. Methods: Seventy patients recruited from a private medical centre met the diagnostic criteria for unilateral Achilles tendinopathy (age, 45.1 ± 12.7 years; weight, 75.00 ± 10 kg; height, 1.75 ± 0.1 m) and were tested based on both Achilles tendons. Seventy patients with a unilateral Achilles tendinopathy ultrasound diagnosis were tested using David’s test. Results: Most (86%) subjects demonstrated Kager’s fat pad asymmetry in relation to the Achilles tendon in the complete passive dorsiflexion in the prone position (David’s sign). No healthy tendons had David’s sign. Conclusions: The presence of asymmetry in Kager’s fat pad in relation to the Achilles tendon during complete passive dorsiflexion is strongly indicative of ultrasound-diagnosed tendinopathy. David’s test demonstrated a sensitivity of 85.71% (95% CI, 77.51% to 93.91%) and a specificity of 100% (95% CI, 100% to 100%), while noting the lack of blinding of the assessors and the uncertainty of the diagnostic measures (95% CI). Asymmetry of the fat pad could potentially serve as a characteristic marker for patients with Achilles tendinopathy.

## 1. Introduction

The Achilles tendon is the strongest tendon of the lower extremities and in the human body. This anatomical component is directly associated with the triceps surae muscle and is modified due to tensile loads by contraction or elongation. This condition makes it susceptible to various injuries, especially those related to overuse; the Achilles tendon is the key to ankle functionality and, therefore, to human locomotion, gait, and posture [1].

Achilles tendinopathy (AT) is a common pathology of great relevance in orthopaedic practice. AT presents the following characteristics: swelling, pain, low functionality, and modified morning stiffness [2]. The medical history record is key to developing the AT clinical diagnosis. The aetiology of AT is multifactorial. Nadeau et al. have found modifications in the tendon thickness and cross-sectional area related to load adaptations and degenerative mechanisms. Moreover, a lack of flexibility in the lower limb; biomechanical factors, such as hyper-pronation, disturbances in blow flow, and a high body mass index (BMI); and a sedentary lifestyle are considered risk factors [1].

Lopes et al. conducted a research review on AT in adult runners and reported a range of 9.1–10.9% for Achilles tendon (AT) incidence and 6.2–9.5% for AT prevalence. In contrast, Albers et al. found lower rates, with a prevalence of 2.35% and an incidence of 2.16% in the adult population (21 to 60 years old) [3]. Among individuals suffering from AT, 75% are between 30- and 49-years-old, and sports activities are a common cause of injury [3].

Quality of life is strongly related to the adequate functioning of the Achilles tendon. Activities of daily living, such as walking, running, going up and down stairs, or even standing, are fundamental to an adequate quality of life.

Several Achilles tendon injuries take place in sports. The incidence of Achilles tendon overuse injuries and complete ruptures has increased in industrialised countries during the last decades [3]. Overuse starts with slight tendon modifications and ends with a structural degeneration component [4]. Loading appears to be one of the most important factors in developing tendinopathies. AT is related to an increased eversion range of motion (ROM) in the rear foot, a shortened maximum lower leg abduction, a decreased ankle joint dorsiflexion velocity, and a decreased knee flexion. In addition, negative differences in ground reaction forces, dynamic plantar pressure, and tibial external rotation have also been reported in patients with AT. Adequate tendon loading is one of the most important elements in ensuring tendon health. If the load is increased or even if the load is decreased, it will play a pathological role and may induce structural modifications in the tendon and pathology [4]. Degeneration is more frequent in the medial portion of the tendon, and this area especially seems to present a reduced vascular component [5]. Imaging assessment techniques are recommended for musculoskeletal disorder assessments [6].

Structural modifications have been identified in individuals with AT. Shaikh et al. [7] showed an increased thickness in runners with AT. Arya and Kulig reported an increase in the cross-sectional area (CSA) in tendinopathic tendons. In addition, Docking and Cook observed a greater Achilles CSA compared to that in healthy individuals [8].

Ultrasound imaging (USI) has been used to gain further insight into assessing the tendon length, thickness, and CSA. USI is considered a non-invasive, safe, and cost-effective technique that provides a complete evaluation of the tendon and related structures and can be performed during tendon activity with a non-invasive, relatively inexpensive, and portable techniques, which may provide a complete assessment of the morphology and size of the Achilles tendon [9].

Healthy Achilles tendons show well-organised, parallel collagen fibres with bright hyperechoic bands and dark hypoechoic bands [10]. Sharma and Maffulli reported areas of disorganisation of unstructured non-parallel collagen fibres and a thickened, hypoechoic portion in the tendons of individuals with AT [11].

Previous research has reported moderate-to-good reliability of the Achilles tendon ultrasound assessment [12,13]. B-mode ultrasound is considered superior to magnetic resonance imaging for assessing structural changes in the AT [13]. Providing information on tendon structure, with data of high clinical interest, such as thickness or CSA, is becoming of interest to clinicians and researchers and is related to diagnosis and observing the evolution of different treatments [13].

Kager’s fat pad (KFP) is a mass of adipose tissue located posterior to the ankle joint complex, related to the Achilles tendon posteriorly, the crural fascia and flexor hallucis longus (FHL) muscle anteriorly, and the posterior calcaneal tuberosity at its base inferiorly [14]. The part associated with the FHL helps move the bursal wedge during plantar flexion, the part associated with the Achilles protects the blood vessels coming into the tendon, and the bursal wedge is thought to protect against pressure changes in the bursa. The close relationship of the KFP to the Achilles tendon has been shown to have a protective effect on the blood vessels crossing the KFP and the tendon itself [15].

The pressure changes within the KFP areas may contribute to AT or fascia cruris tears [16,17]. Similar studies have been performed in other areas of the body to evaluate the infrapatellar fat pad of the knee, suggesting that it plays a role in stabilising the patella [18].

KFP pressure modifications have been checked, and there is controversy about whether movement limitations result in increased pressure loading on the retro-calcaneal bursa and medial portion of the Achilles tendon [19,20]. Hence, this study aims to establish the asymmetry between the KFP in AT as a key point for clinical assessments.

Several orthopaedic tests are applied in clinical assessments to check for passive and active ankle ROM, but there is no clinical test for assessing the KFP and its relationship with the Achilles tendon. This study aimed to check the diagnostic accuracy results of a new test to identify AT.

## 2. Materials and Methods

Seventy subjects met the ultrasound diagnostic criteria for unilateral AT based on tendon thickness, echogenicity, and vascularisation, which was considered confirmatory of pathology in this region [1]. The participant mean age was 45.1 ± 12.7 years, with a mean weight of 75.00 ± 10 kg and height of 1.75 ± 0.1 m. The Epidat 4.2 program (Consellería de Sanidade, Xunta de Galicia, España; Organización Panamericana de la salud (OPS-OMS) was used to calculate the sample size.

### 2.1. Study Design

An observational study was carried out in 2021, according to the Standards for Reporting Diagnostic Accuracy (STARD) guidelines [21].

### 2.2. Ethics

The Research and Ethics Committee of HULP (Madrid, Spain; record number: 2828 A) approved the study. Informed consent forms were completed and signed by all of the study participants. The Biomedical Declaration of Helsinki of 1975, revised in 2013, was respected throughout the research. According to point 23 of this declaration, approval from the local institutional review board (IRB) or another appropriate ethics committee must be obtained before undertaking the research to confirm the study meets national and international guidelines.

The IRB approved this study based on participants with an ultrasound diagnosis of AT. All USI images were obtained by the same therapist (JVS). Of these, the subjects with AT confirmed through USI were considered for inclusion in this study. No funding resource was obtained in this study.

### 2.3. Clinical Assessment

Seventy subjects who met the ultrasound diagnostic criteria for unilateral AT were included in the study. It should be noted that the use of pre-selected patients from a private clinical centre specialising in sports injuries may introduce bias into the research. The patients were recruited as a consecutive sample from this centre.

The inclusion criteria were as follows: 18–65-years-old, had pain or soreness in the mid-portion of the Achilles tendon for at least 3 months, tendinopathy confirmed via ultrasound, had a visual analogue scale pain intensity score of at least 3 out of 10 points, and had no received any intervention or treatment. Exclusion criteria were patients with skin diseases or systematic disorders, previous fractures, and lower limb pathology in the last 12 months and KFP pathology. The patients were evaluated for asymmetry of the fat pad with reference to the Achilles tendon in maximum passive dorsiflexion with the knee extended in the prone position. Since the patients were passive dorsiflexed by the therapist, the therapist could also sense the tightness of AT or painful reactions. This assessment was performed by the same therapist (DRS) with 20 years of clinical experience. Participants did not receive treatment before the clinical and ultrasound assessment. The authors proposed David’s test denomination for this evaluation.

**Figure 1:**
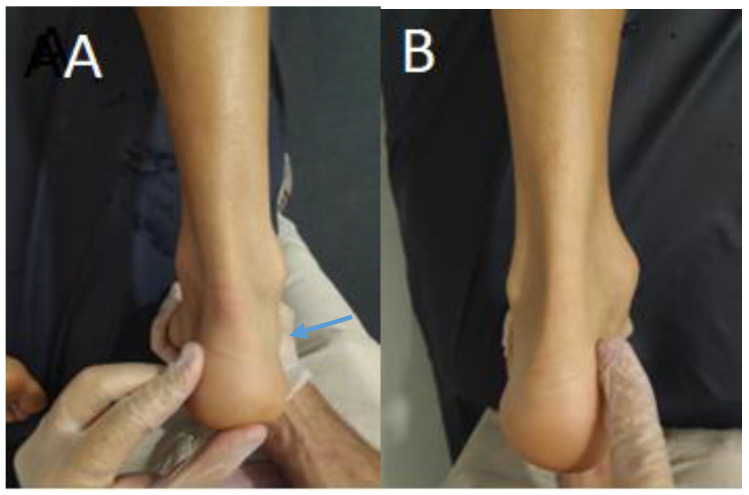
(**A**) Asymmetric KFP (blue arrow) in maximum passive ankle dorsiflexion. (**B**) Symmetric KFP in maximum passive ankle dorsiflexion.

If this assessment was positive, there were differences between the lateral and medial sides of the Achilles tendon. The arrow in Figure 1A marks an asymmetric belly (KFP) in the medial side of the Achilles tendon. The authors proposed David’s sign for this asymmetric belly.

If the test was negative, there were no differences between the lateral and medial sides of the Achilles tendon (as shown in Figure 1B).

### 2.4. Ultrasound Imaging Assessment

The UI examination was performed with a LogiQ P7 (GE Healthcare; Chalfont Saint Giles, UK) with a 4 to 13 MHz linear transducer (38 mm footprint with an L6-12-RS type) for musculoskeletal structures and was employed to develop the ultrasound recordings in B-Mode. All measurements were performed by a single operator (JVS) on the same day and before the clinical assessment in a different room. The rater had 10 years of experience in ultrasound assessment. The ultrasound evaluation was performed in the prone position, with both feet hanging over the end of the table. In this position, the Achilles tendon enthesis in the calcaneus was located using ultrasound.

**Figure 2:**
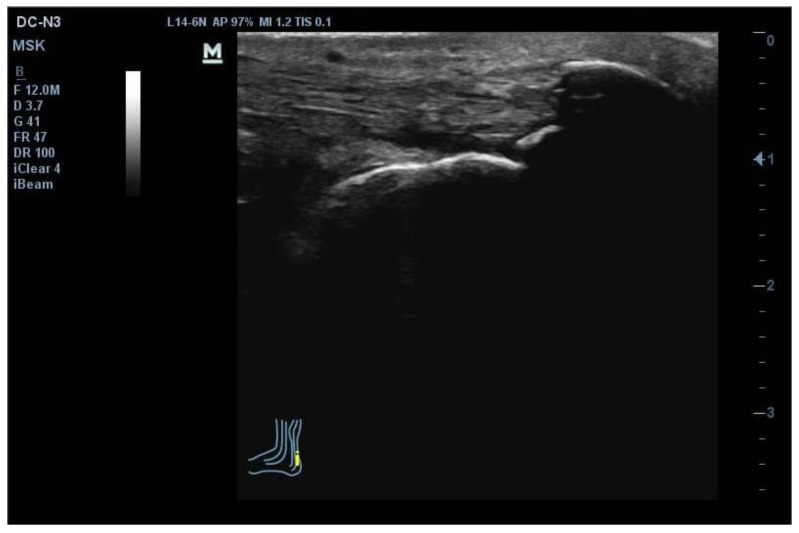
Achilles complex ultrasound assessment image. The blue and yellow lines are the anatomical structure and the ultrasonographic linear transducer position.

Ultrasound images were obtained to detect the presence of AT based on tendon thickness, echogenicity, and vascularisation, which was considered confirmatory of pathology in this region [1]. For measuring Achilles tendon integrity via quantitative ultrasound, patients were placed in a prone position with the foot inside the stretcher with passive dorsal flexion [22]. The probe was placed in the central region of the medial gastrocnemius, oriented along the medial longitudinal axis determined based on the soleus fibres (Figure 2).

### 2.5. Statistical Analysis

The association between ultrasound evaluation, as the gold standard, and the findings obtained in the clinical examination were evaluated using McNemar’s statistical test for paired proportions, and sensitivity and specificity were estimated as binomial proportions with exact 95% confidence intervals (CIs). The results obtained were considered statistically significant for *p*-values less than 0.05. A priori analyses indicated that 60 records would allow for an estimation of the statistical sensitivity of David’s sign (concerning the ultrasound evaluation) within a margin of error of 5.5% if the true sensitivity of David’s sign was 95% or higher (i.e., the maximum mean width for a 95% confidence interval for sensitivity would be 5.5%). SPSS 22.0 software (IBM SPSS Statistics for Windows, IBM Corp., Armonk, NY, USA) was used to analyse the data.

## 3. Results

Of the 70 patients with an AT ultrasound diagnosis that met the diagnostic criteria for unilateral AT over 6 months, all were tested using David’s test, and 60 of the 70 (85.7%) demonstrated KFP asymmetry related to the Achilles tendon in maximum passive ankle dorsiflexion (David’s sign). None of the healthy tendons were positive for the test. The control group consisted of the other healthy legs of the studied patients. No adverse events from performing the index test or the reference standard were observed (Table 1).

Many patients had no symmetry in the anatomic region posterior ankle due to the calcaneus position, which is closely related to Achilles tendon performance and health.

In positive cases, the KFP had no symmetry related to the Achilles tendon after passive dorsiflexion in the prone position. Patient records considered positive for the physical examination directly referred to this fact. David’s sign is an asymmetry of the KFP position in relation to the Achilles tendon during the ankle ROM and maximum passive dorsiflexion in the prone position.

The presence of asymmetry of the KFP related to the Achilles tendon in complete passive dorsiflexion in association with the presence of tendinopathy made the clinical assessment finding significantly related to the results obtained with the ultrasound evaluation developed (McNemar’s statistical test for association, *p* = 0.01). The sensitivity achieved using David’s test was 85.714% (95% CI, 77.51% to 93.91%), with a specificity of 100% (95% CI, 100% to 100%) (Table 2).

## 4. Discussion

The diverse characteristics of AT are of particular interest for the effective management of patients, encompassing aspects, such as diagnosis, treatment, and the clinical course. AT can affect individuals across various demographics and is associated with distinct lifestyle, occupational, and sports-related factors.

Various structures can be modified in AT. Some are located close to the tendon, such as the plantar fascia, fat heel pad, intrinsic foot muscles, or extrinsic foot muscles, while others are in more distant anatomical locations, like the abdominal muscle core. Patients with AT have been observed to exhibit a decreased thickness of the transverse abdominis, internal oblique, and external oblique, along with an increased inter-abdominal distance. These modifications in the abdominal stabilising musculature are particularly relevant as they are associated with alterations in distal structures, such as the calcaneus, KFP, and Achilles tendon, which are closely linked to motor control and, therefore, injury prevention.

Adequate muscular conditions of the transverse abdominis, internal oblique, and external oblique will be factors of interest in activities, like walking, running, going up and down stairs, and standing. These activities are intimately related to an improved level of quality of life [21,22,23,24,25,26,27,28].

Changes in foot biomechanics can lead to the development of other foot problems, such as degenerative arthritis or sinus tarsi syndrome. Additionally, alterations may arise in association with neurological pathological foot components or the various capsuloligamentous elements of the foot. The static and dynamic position of the KFP appears to play a crucial role in protecting the Achilles tendon [27].

Previous research has reported increased tendon tension and decreased tendon stiffness in individuals with AT [27]. Kongsgaard et al. demonstrated that stiffness remained unaffected in control tendons but declined in tendinopathic tendons [23]. These modifications seem to be interrelated and direct consequences of changes in the cellular tendon structure, as indicated by the relationship between loads and AT. Both increased and decreased loads play crucial roles in the development of AT [18].

It has been proposed that the KFP provides mechanical assistance in the insertion of the Achilles tendon by entering the retrocalcaneal bursa during plantar flexion. This positioning of the fat optimises the lever arm of the tendon. The KFP, along with the bursa, forms a highly mobile wedge of fat closely related to the calcaneus, allowing it to move both in and out of the bursa. During plantar flexion, the calcaneus moves passively and actively into the retrocalcaneal bursa. Despite its significance, this structure has received limited attention in research, and its biomechanical contributions are not well understood. It remains unclear how alterations in the specific mechanics of the KFP could lead to pathology in the anatomical components posterior to the ankle joint [19].

There are other clinical assessment tests for AT, such as pain on palpation of the tendon (sensitivity 84%, specificity 73%) and the subjective reporting of pain 2–6 cm above the insertion into the calcaneum (sensitivity 78%, specificity 77%) [29].

Ultrasound M-mode and ultrasound elastography assessments could be essential tools for researching the Achilles tendon [30,31,32], KFP, retrocalcaneal bursa, and deep soleal fibres, providing additional imaging analyses. Previous research has revealed structural modifications in the Achilles complex and identified changes in patients with AT. Shaikh et al. [7] and Arya and Kulig [8] demonstrated modifications in tendon thickness and CSA in athletes diagnosed with AT. Padhiar et al. [33] reported that patients with AT exhibit a decrease in the pennation angle of soleus muscle fibres, which is directly related to a strength deficit in the evaluated participants.

If David’s sign proves to be an adequate assessment, the need for obtaining other imaging studies could potentially be reduced. However, this test might be considered a preliminary step before ultrasound assessment, given its potential bias towards a pre-selected AT sample. Our review of subjects with AT and available ultrasound clinical assessments revealed a strong correlation between KFP asymmetry and tendon pathology, as well as fluid presence within the tendon sheath. It is important to note that future research should focus on developing intra and inter-observer reliability to address this as a limitation.

B-mode ultrasound assessment is considered essential for detecting subtle modifications within the tendon or tendon sheath and for identifying structural alterations in AT. These changes, which may be related to early AT conditions, might not yet have progressed to the point where other clinical associations yield positive results [11].

Assessing AT [34] plays a crucial role in determining the course of treatment and selecting appropriate therapeutic tools [35,36,37,38]. Recognisable clinical signs may be present at various stages, guiding the use of different treatment approaches. While we acknowledge several limitations in our study, this test remains interesting and could aid clinicians in safeguarding the structure of their patients’ Achilles tendons.

Clinical assessment tests are essential for clinicians to gain a better understanding of the status of the Achilles tendon in patients. David’s test could be considered a preliminary assessment before performing an ultrasound examination.

### Limitations

Some limitations related to this research must be considered. The conditions affecting the shape of the ankle should be considered as limitations of this method (e.g., lymphedema, posttraumatic oedema, and posttraumatic altered bone shape in the area of the foot and ankle).

The study subjects were collected from the same country and region, and all participants were actively engaged in sports activities. It would be intriguing to test different populations, including individuals with a larger BMI and increased fat distribution around the ankle area. It is worth noting that all measurements were conducted in non-weight-bearing situations. An important bias to consider is that the patients enrolled in the study already had a confirmed diagnosis of AT, which means that the assessors were not blind to the condition.

Unfortunately, the research did not assess inter-observer or intra-observer reliability. Moving forward, it would be valuable to include such assessments to enhance the study’s validity. Additionally, given the varying loads that the Achilles tendon can encounter based on the specific physical activities performed, analysing different populations of athletes is of great interest. This would help to develop appropriate diagnostic and therapeutic approaches tailored to the specific needs of each population.

## 5. Conclusions

The asymmetric position of the KFP (David’s sign) in relation to the Achilles tendon during the maximum passive dorsiflexion of the ankle ROM appears to be strongly associated with the presence of AT. In our experience, although the assessors were not blinded and the diagnostic measures had some uncertainty (95% CI), David’s sign, when combined with a comprehensive clinical examination, could potentially reduce the need for obtaining imaging assessments. However, if doubts persist about the clinical diagnosis after the clinical examination, an imaging assessment can be used as a confirmatory test.

To enhance our understanding of this sign and its biomechanical and clinical associations, future research should aim to improve knowledge and gather more data.

## Figures and Tables

**Table 1:** A 2 × 2 table for sensitivity and specificity analysis.

	Ultrasound Assessment		
Test	Positive	Negative	Sum Value
Positive	60	0	60
Negative	10	70	80
Sum Value	70	70	140

**Table 2:** Statistical diagnostic accuracy measures data.

Statistical Diagnostic Accuracy Measures	
Sensitivity	85.71% (95% CI 77.51% to 93.91%)
Specificity	100% (95% CI 100% to 100%)
Positive predictive value	100% (95% CI 100% to 100%)
Negative predictive value	87.5% (95% CI 80.25% to 94.74%)
False positive rate	0%
False negative rate	14.28%
Youden’s index	0.875
Provided properly classified	92.85%
Provided unproperly classified	7.14%
The number needed to diagnose (Bayesian calculations)	1.167
The number needed to misdiagnose (Bayesian calculations)	14

CI: confidence interval.

## Data Availability

Not applicable.
